# Changes in the Prevalence of HBsAg and HBeAg: a Study of 8696 Parturients in a Well Vaccinated Area

**DOI:** 10.1038/s41598-017-01234-0

**Published:** 2017-04-27

**Authors:** Chen-Hsuan Wu, Te-Yao Hsu, Fu-Tsai Kung, Chan-Chao ChangChien, Ching-Chang Tsai, Sheng-Nan Lu

**Affiliations:** 1 Graduate Institute of Clinical Medical Sciences, College of Medicine, Chang Gung University, Taoyuan, Taiwan; 2 Department of Obstetrics and Gynecology, Kaohsiung Chang Gung Memorial Hospital and Chang Gung University College of Medicine, Kaohsiung, Taiwan; 3 Division of Hepatogastroenterology, Department of Internal Medicine, Kaohsiung Chang Gung Memorial Hospital and Chang Gung University College of Medicine, Kaohsiung, Taiwan

## Abstract

To elucidate the impact of a hepatitis B (HB) vaccination program on the prevalence of HB surface antigen (HBsAg) and HB envelope antigen (HBeAg) as well as the success rate of HBeAg clearance among parturients, we collected data on parturients who gave birth between 2000 and 2010, and recorded the HB status postpartum of those with positive HBeAg before birth. A total of 8696 parturients were enrolled, of whom 113 with prenatal positive HBeAg were invited back. The prevalence of HBsAg decreased over the study period, particularly in the vaccinated cohort, while there was no change in the prevalence of HBeAg. Foreign parturients had a higher HBeAg-positive rate and delayed HBeAg clearance, and those with a higher body mass index (>24 kg/m^2^) had earlier HBeAg clearance (51.9% vs. 23.9%, p = 0.005). Only 30% of the subjects who were positive for HBeAg before birth became negative 5 years after delivery. In conclusion, the downward trend in HB infection with more significance among vaccinated parturients reflects effective prevention and the impact of universal HB immunization. Nonetheless, aggressive follow-up is necessary for parturients who are persistently positive for HBeAg postpartum, as well as developing different public health policies for foreign parturients from endemic areas.

## Introduction

There are almost 360 million chronic carriers of hepatitis B (HB) virus (HBV) worldwide, with a higher prevalence in Asian countries^1^. In Taiwan, HBV infection is an important public health issue due to its association with hepatocellular carcinoma (HCC), the second leading cause of cancer death of Taiwan, and chronic liver disease^2^. Moreover, HBsAg-positive parous women have been reported to have a significantly high mortality rate due to both liver and non-liver-related causes^3^.

A high prevalence of HB envelope antigen (HBeAg) among pregnant women positive for HB surface antigen (HBsAg) has been reported to result in a high incidence of HBV infection in newborns through mother-to-infant transmission^4^. In July 1984, Taiwan launched a nationwide HB vaccination program for infants born to HBV carrier mothers, primarily to interrupt vertical transmission. Two years later, the vaccination program extended the coverage to all newborns, and also gave hepatitis B immune globulin (HBIG) injections to the newborns of HBeAg-positive mothers within 24 hours after birth. Thus Taiwan was the first country to introduce an HB vaccination program in the world^5^. The program as then further extended to cover all preschool children in 1987, primary-school children in 1988, and middle-school children in 1989, resulting in a high national coverage rate in the targeted cohort of 92.8% by 2002^6^.

Early HBeAg seroconversion in the immune clearance phase has been shown to indicate a favorable outcome in the natural course of chronic HBV infection. In contrast, late HBeAg clearance has been associated with the progression of chronic hepatitis and a poor long-term outcome such as liver cirrhosis and HCC^7–9^. Previous studies have reported a decrease in the prevalence of HBeAg among pregnant women in Taiwan during the 1990s^10, 11^; however, trends in those vaccinated after the introduction of the vaccination program have yet to be reported. Since the vaccinated cohort are now of child-bearing age, we aimed to investigate the effect of the universal HBV vaccination program in Taiwan on this cohort, including the trend of HBeAg prevalence during the 2000s. Moreover, during the past decade an increasing number of foreign spouses have immigrated to Taiwan, mostly from China and other Southeast Asian countries with a high prevalence of HBV infection^12, 13^. Therefore, we also aimed to investigate the status of HBV infection among Taiwanese pregnant women and those from other Asian countries in order to explore the potential impact of foreign immigration on public health in Taiwan.

We conducted this large-scale 10-year retrospective analysis to explore the trends of the prevalence of HBsAg and HBeAg in pregnant women, and also a follow-up study for the mothers who were HBeAg-positive to evaluate the rate of postpartum HBeAg seroconversion as well as the associated factors. We hope the results can provide data on the impact of the universal hepatitis B vaccination program in Taiwan, and also provide a reference to improve the efficacy of such programs.

## Results

### Retrospective study

From January 2001 to April 2010, we recruited 8696 pregnant women including 8258 Taiwanese women and 438 foreign spouses from China and other Southeast Asian countries. We excluded 426 (4.9%) who had no data on birth date (Table 1). The prevalence rate of positive HBsAg decreased in each year over the 10-year study period (*p* < 0.001). By stratifying the mothers by birth date into four groups under different coverage strategies of HBV vaccination as before June 1979, July 1979 to June 1984 (5 years before universal immunization to newborns born to HBeAg-positive mothers), July 1984 to June 1986, and after July 1986 (nationwide vaccination to all newborns), the HBsAg-positive rates were 15.9%, 12.4%, 7.8%, and 2.6%, respectively (*p* = 0.001). The subjects born after the universal vaccination program had been implemented (July 1986) had a much lower seropositive rate than those born before the program had been launched (June 1984) (*p* < 0.001). However, the carrier rate had already started to decrease in the women born 5 years before the introduction of the HB vaccination program (July 1979 to June 1984). With regards to nationality, we did not find any statistical difference in the HBsAg carrier rate between Taiwanese and other Asian women (14.5% vs. 12.6%, *p* = 0.249).

**Table 1:** Number of parturients and prevalence rate of positive HBsAg from 2000 to 2010.

Factors		Number of subjects	Number HBsAg(+)	Percentage HBsAg(+)	P_1_	P_2_
Total		8696	1256	14.4%		
Calendar year of HBsAg test	2000	928	147	15.8	0.001	0.001
	2001	1561	256	16.4		
	2002	1558	206	13.2		
	2003	597	112	18.8		
	2004	403	59	14.6		
	2005	513	64	12.5		
	2006	628	94	15.0		
	2007	718	103	14.3		
	2008	707	75	10.6		
	2009	729	97	13.3		
	2010	354	43	12.1		
Birthday	Before June 1979	6905	1099	15.9	<0.001	<0.001
	July, 1979 to June 1984	1186	147	12.4		
	July, 1984 to June 1986	103	8	7.8		
	After July, 1986**	76	2	2.6		
Nationality	Native	8258	1201	14.5	0.249	
	Other Asian countries	438	55	12.6		

*P_1_. Pearson’s chi-square test; P_2_. chi-square test for a linear trend.

**Stratifying their birth of dates into four groups based on the time when the HBV vaccination program was launched in Taiwan.

A total of 263 HBeAg-positive mothers with 353 deliveries were identified from 1256 HBsAg-positive mothers with 1595 deliveries. No trend was identified in the positive rates of HBeAg during the 2000s (Table 2). Even so, the positive rates of HBeAg did decline with age, from 42.0% in women aged less than 25 years to 9.9% in those aged 35 years or older (*p* < 0.001). Neither the number of pregnancies nor parity were significantly associated with the positive rate of HBeAg. Moreover, women born in Taiwan had a significant lower HBeAg-positive rate than those from other Asian countries (21.3% vs. 40.6%, *p* < 0.001). In multiple logistic regression analysis, age and nationality were still independently associated with HBeAg-positive rate (Table 3). Compared to the women aged 35 years or older, those parturients aged <25, 25–29 and 30–34 years had higher positive HBeAg rate with odds ratios (ORs) (95% confidence intervals (CIs)) of 5.97 (3.52–10.13), 3.49 (2.30–5.31), and 2.18 (1.44–3.32), respectively. The OR of a positive HBeAg rate was 1.74 (1.03–2.92) for the women from other Asian countries compared to Taiwanese women (*p* = 0.037).

**Table 2:** Number and prevalence of positive HBeAg rate among HBsAg carrier parturients who delivered from 2001 to 2010.

Factors		Number HBsAg(+)^a^ n = 1595	Number HBeAg(+)^b^ n = 353	Percentage HBeAg(+) 22.1%	P_1_	P_2_
Calendar year of delivery	2001	232	62	26.7	0.647	0.249
	2002	258	56	21.7		
	2003	190	35	18.4		
	2004	78	22	28.2		
	2005	113	24	21.2		
	2006	167	38	22.8		
	2007	150	32	21.3		
	2008	144	30	20.8		
	2009	149	32	21.5		
	2010	114	22	19.3		
Age (years)	<25	119	50	42.0	<0.001	<0.001
	25–29	512	145	28.3		
	30–34	652	127	19.5		
	≥35	312	31	9.9		
Number of pregnancies	1	348	78	22.4	0.386	0.391
	2	655	132	20.2		
	3	382	94	24.6		
	>3	210	49	23.3		
Parity	1	510	111	21.8	0.613	0.398
	2	823	179	21.7		
	3	219	50	22.8		
	>3	43	13	30.2		
Nationality	Native	1526	325	21.3	<0.001	
	Foreign	69	28	40.6		

^*^P_1_. Pearson’s chi-square test; P_2_. chi-square test for linear trend.

^a^Number of deliveries from 1256 HBsAg-positive mothers.

^b^Number of deliveries from 263 HBeAg-positive mothers.

**Table 3:** Multiple logistic regression analysis for factors associated with HBeAg among HBsAg-positive parturients.

Factor	Comparison	OR (95% C.I.)	P value
Age (years)	<25 vs. >=35	5.97 (3.52–10.13)	<0.001
	25–29 vs. >=35	3.49 (2.30–5.31)	<0.001
	30–34 vs. >=35	2.18 (1.44–3.32)	<0.001
Nationality	Other Southeast Asian countries vs. Taiwanese	1.74 (1.03–2.92)	0.037

### Follow-up study

A total of 133 women of the 263 HBeAg-positive mothers responded to our request to return for a postpartum follow-up study. After excluding 14 subjects who had received antiviral therapy, the remaining 119 were recruited for analysis. The mean age of these mothers at follow-up was 35.2 ± 4.7 years, and the mean duration from the first diagnosis of being HBeAg-positive during pregnancy to returning for the follow-up study was 5.1 ± 3.2 years. Thirty-six (30.3%) of the women tested negative for serum HBeAg with HBV DNA levels less than 2000 IU/ml, and were regarded as having successful HBeAg clearance. In addition, among those mothers who were persistently positive for HBeAg, 86.7% had an HBV DNA level higher than 2000 IU/ml (not shown in the tables). In univariate analysis comparing factors related to HBeAg clearance, body mass index (BMI), fatty liver and nationality had a trend of association with successful HBeAg clearance (Table 4). Furthermore, in multiple logistic regression analysis for these associated factors, only BMI was statistically significant, showing a delayed HBeAg clearance in the women with a BMI < 24 kg/m^2^ (OR 3.43, CI 1.40–3.38, *p* = 0.007).

**Table 4:** Univariate analysis for successful HBeAg clearance and associated factors among parturients who were HBeAg-positive before delivery.

Variable	Comparison	HBeAg clearance		P value
		Failure^b^ n = 83 (69.7%)	Success^c^ n = 36 (30.3%)	
Duration	Years	4.8 ± 3.2	5.8 ± 2.9	0.101
Age	Years	34.7 ± 4.8	36.4 ± 4.2	0.070
Nationality	Foreign	7 (100)	0 (0)	0.100
	Native	76 (67.9)	36 (32.1)	
Number of parity	1	27 (71.1)	11 (28.9)	0.928
	2	43 (68.3)	20 (31.7)	
	≥3	13 (72.2)	5 (27.8)	
BMI	≤24 kg/m^2^	70 (76.1)	22 (23.9)	0.005
	>24 kg/m^2^	13 (48.1)	14 (51.9)	
Fatty liver	N	48 (77.4)	14 (22.6)	0.057
	Y	35 (61.4)	22 (38.6)	
HBV	B	59 (72.8)	22 (27.2)	0.177
genotype^a^	C	22 (88.0)	3 (12.0)	

^a^Subjects with detectable HBV DNA were included.

^b^HBeAg(+) or HBeAg(−) but HBV DNA > 2000 IU/ml.

^c^HBeAg(−) and HBV DNA < 2000 IU/ml.

## Discussion

In this study, we found that the prevalence of HBsAg had already begun to decrease in the women born as early as 5 years before the introduction of the national HB vaccination program in Taiwan, and there was an even more dramatic decrease in the vaccinated cohort. We also found that the HBeAg-positive rate in the HBsAg-positive parturients was consistently around 22% over the study period, with a decrease in the rate with age. Foreign parturients seemed to have a higher HBeAg-positive rate as well as delayed HBeAg clearance compared to Taiwanese parturients. Furthermore, the women with a BMI < 24 kg/m^2^ also had delayed HBeAg clearance compared to those with a BMI > 24 kg/m^2^. Among the women positive for HBeAg identified during the prenatal period, about 30% were negative for serum HBeAg with HBV DNA levels <2000 IU/ml. However, the HBeAg-positive rate among the HBsAg-positive mothers with a mean age of 35 years as still as high as 69.7% even 5 years after delivery.

In July 1984, Taiwan launched a mass nationwide hepatitis B vaccination program for infants born to HBsAg-positive mothers. Two years later, the program was extended to cover all newborns as well as to give HBIG injections to the newborns with HBeAg-positive mothers^14, 15^. In the current study, the overall prevalence rate of HBsAg in the pregnant women over the past 10 years was 10.6–18.8%, with a decreasing trend in each calendar year. There was an abrupt decrease in the number of parturients in 2003, as the hospital was isolated due to a nosocomial outbreak of severe acute respiratory syndrome (SARS)^16^. We found a dramatic decrease in the HBsAg-positive rate to 2.6% in the vaccinated parturients, suggesting that the universal immunization program was successful in reducing the rate of perinatal infection. Interestingly, we also noted a decrease in the prevalence rate of HBsAg among the group born before the universal vaccination program was launched, suggesting the protective impact of the additional vaccination since 1987 on pre-school and middle school children. Similar results were also reported by a systematic review reporting the prevalence of HBV infection in two time periods (1990 and 2005)^17^. Moreover, owing to efforts on primary strategies to reduce or eliminate the risk of HBV transmission from nosocomial exposure (e.g. screening for safe blood and blood products for transfusion, use of disposable syringes and needles and universal precautions), the risk of hepatitis B infection as well as super-infections with hepatitis D virus among HBV carriers has dramatically decreased, which also had a protective impact on the unvaccinated cohort^18, 19^.

Lu *et al*. and Lin *et al*. reported a decrease in the HBeAg-positive rate by year in the 1990s among HBsAg-positive mothers^10, 11^, and a systematic literature review conducted to estimate age- and region-specific HBeAg prevalence rates for females in the Asia Pacific region also showed a remarkable decrease in HBeAg prevalence from 1990 to 2005, and this was even more pronounced among those aged <9 years^20^. In the current study, the HBeAg-positive rate further declined to 22% in the early 2000s, but remained stationary thereafter. The decrease in positive HBeAg rate in the 1990s may be explained by marked improvements in the living environment and economy in Taiwan, while these improvements may not have been as pronounced in the 2000s.

HBeAg clearance has been reported to be both a critical serological endpoint for patients with chronic hepatitis B and also an important consideration when choosing the treatment to achieve this goal^21^. The reported factors associated with early HBeAg clearance include age, parity, HBV genotype, pretreatment low serum HBV DNA level, molecular virological factors as well as the degree of both biochemical and histologic activity^22–25^. Lin *et al*. reported that the positive HBeAg rate in Taiwanese parturients from 1985 and 1990 decreased from 49.4% in those aged 21–25 years to 20% in those aged >35 years^11^. Lu *et al*. also reported a similar decline with advanced age in the 1990s^10^. In the current study, the HBeAg-positive rate among the HBsAg-positive mothers still declined with increasing age in the past 10 years, and it is encouraging that the prevalence was much lower than in the equivalent group in the 1990s.

Several studies have reported that immigrants from endemic areas with a higher HBV prevalence and later implementation of vaccination programs can be a challenge for public health policies of the countries to which they move^26–28^. We found that the foreign parturients had higher HBeAg prevalence and delayed HBeAg clearance rates compared to the Taiwanese mothers. Despite World Health Organization (WHO) recommendations that HBV vaccines should have been incorporated into routine childhood immunization programs by 1997, and that the primary strategy to control HBV infection is universal infant immunization, some countries have not been able to afford to implement such measures as early as recommended^29, 30^. Chu *et al*. reported a change in the epidemiology of hepatitis B related to migration by comparing six northern and north-western European countries^31^. Their results showed evident differences in the HBsAg prevalence rate between the migrants and general population, which the authors concluded was because most of the migrants acquired the infection in early childhood in their country of birth. In Taiwan, most foreign mothers come from China and Vietnam. China launched a nationwide HBV vaccination program in 2002^32^, and Vietnam’s National Expanded Program on Immunization (NEPI) introduced hepatitis B vaccines in 1997. However, a number of infant deaths in 2006 were linked to the HBV vaccine in Vietnam, hence its coverage rate was only 20% by 2008^33^. This may explain why we found higher HBeAg prevalence and delayed HBeAg clearance rates in the foreign parturients than in those born in Taiwan. This issue cannot be ignored, and different strategies should be considered for HBV infection surveillance in the migrant population^34^.

Although nationality, BMI, fatty liver and HBV genotypes were all associated with HBeAg clearance, BMI was the only statistically significant factor in this study. Although the impact of BMI on the natural history of chronic hepatitis B infection still remains unclear, several studies have demonstrated an inverse relationship between BMI and metabolic syndrome and HBsAg serum clearance^35–37^. Chiang *et al*. reported a relationship between extreme obesity or central obesity and a low prevalence of high HBV viral load in patients seropositive for HBeAg^38^. Larger longitudinal studies are required to further clarify the association and mechanism between obesity and the natural course of HBV infection.

Genotypes B and C are the predominant HBV strains in far-eastern Asian countries. Genotype C has been associated with more severe liver disease and delayed spontaneous HBeAg seroconversion than genotype B in both adults and children in Taiwan^39, 40^. The HBV DNA of the HBeAg-positive mothers was checked during the follow-up study, however not all parturients in Taiwan undergo routine HBV DNA testing before delivery. Therefore, we could not analyze those with postpartum successful HBeAg clearance and undetectable HBV DNA, which may be a limitation of this study.

In conclusion, the downward trend in HB infection among parturients, and particularly in the vaccinated cohort, reflects effective horizontal prevention as well as the impact of the universal HB immunization program. It is worth noting that among the parturients who were positive for HBeAg before birth, up to 70% did not achieve serum HBeAg clearance even 5 years after delivery. Therefore, aggressive follow-up for these parturients and also developing different public health policies for foreign parturients from endemic areas to prevent subsequent complications caused by persistent chronic HBV infection are necessary.

## Methods

All experiments were performed in accordance with the relevant guidelines and regulations, and informed consent was obtained from all participants for the chart review and to use blood samples to check hepatitis B status. This study was approved by the Institutional Review Board of Chang Gung Memorial Hospital (CGMH), Taiwan, and it was also funded by a grant from CGMH (CMRPG8A0031) and the Ministry of Science and Technology of Taiwan (NMRPG8F0261).

### Retrospective study

All parturients who delivered between January 2001 and April 2010 at the Department of Obstetrics of CGMH in southern Taiwan were recruited. Serum HBsAg and HBeAg were tested routinely at the prenatal clinic in all parturients at a gestational age between 28 to 32 weeks. Therefore, some of the parturients who delivered in early 2001 may have been tested for HBV at the end of 2000. We retrospectively obtained data including date of birth, nationality, parity, date of delivery, and the results of HBsAg and HBeAg tests during pregnancy from their medical records. In order to compare the HBsAg and HBeAg positive rates over time and according to four different coverage strategies of HB vaccination (before June 1979, from July 1979 to June 1984 (5 years before universal immunization was given to infants born to HBeAg-positive mothers), July 1984 to June 1986, and after July 1986 (when nationwide vaccination was given to all newborns), we performed analysis stratified by age, parity, date of birth, and relevant time periods.

### Follow-up study

In April 2011, we invited 263 mothers confirmed to be HBeAg-positive during pregnancy from 2001 to 2010 to return to check their hepatitis status. All of the women provided informed consent, and all provided blood samples to measure the level of HBeAg, titer of HBV DNA and genotype of the HBV. HBeAg was analyzed using a microparticle enzyme immunoassay (AxSYM, Abbott GmbH & Co. KG, Wiesbaden, Germany), and HBV DNA was detected by real-time PCR (TaqMan HBV analyte-specific reagent, ASR; Roche Molecular Systems, Inc., Branchburg, NJ). HBV genotypes were determined using a restriction fragment length polymorphism on the surface gene (between nucleotide positions 256 and 796), which was amplified by PCR using nested primers as described in our previous study^41^. Women negative for HBeAg who were previously HBeAg-positive were defined as having HBeAg clearance^42^. We defined the subjects who were negative HBeAg and an HBV DNA level <2000 IU/ml as the “success” group, and we excluded those who had received antiviral therapy for HB.

### Statistical analysis

Continuous and categorical data were presented as mean, standard deviation, and percentage. Comparisons between groups and linear trends between variables were analyzed using the chi-square test and chi-square test for trends, respectively. In univariate analysis, Fisher’s exact test was also used to examine group differences for categorical variables. Independent factors possibly associated with HBeAg prevalence and clearance were analyzed using multiple logistic regression models. A *p* value of <0.05 was considered to be statistically significant.
